# Construction of Diagnostic Model for Regulatory T Cell-Related Genes in Sepsis Based on Machine Learning

**DOI:** 10.3390/biomedicines13051060

**Published:** 2025-04-27

**Authors:** Xuesong Wang, Zhe Guo, Xinrui Wang, Zhong Wang

**Affiliations:** Beijing Tsinghua Changgung Hospital, School of Clinical Medicine, Tsinghua University, Beijing 100084, China; wxs1103@mail.tsinghua.edu.cn (X.W.);

**Keywords:** sepsis, regulatory T cells, machine learning, biomarkers

## Abstract

**Background**: Sepsis is a complex syndrome caused by a severe infection that occurs with a severe inflammatory response. Regulatory T cells (Tregs) have immunosuppressive effects and play a crucial role in modulating the immune response. There-fore, the number of Tregs is significantly increased in sepsis patients. **Methods and Results**: This paper aims to identify Tregs associated with the diagnosis of sepsis. For this purpose, transcriptional data from the GEO database for sepsis and its controls were downloaded and subjected to differential expression analysis. Immuno-infiltration analysis of the obtained DEGs revealed that Tregs were significantly different in sepsis and its controls. To further explore the cellular landscape and interactions in sepsis, single-cell RNA sequencing (scRNA-seq) data were analyzed. We identified key cell types and their interactions, including Tregs, using cell–cell communication analysis tools such as CellChat. This analysis provided in-sights into the dynamic changes in immune cell populations and their communication networks in sepsis. Thus, we utilized multiple machine learning algorithms to screen and extract Treg-related genes associated with sepsis diagnosis. We then performed both in-ternal and external validation tests. The final diagnostic model was constructed with high diagnostic accuracy (accuracy of 0.9615). Furthermore, we verified the diagnostic gene via a qPCR experiment. **Conclusions**: This paper elucidates the potential diagnostic targets associated with Tregs in sepsis progression and provides comprehensive understanding of the immune cell interactions in sepsis through scRNA-seq analysis.

## 1. Introduction

Sepsis is a critical illness caused by severe infection with high morbidity and mortality. The development and progression of sepsis involve an inflammatory response triggered by complex interactions between microorganisms and the host [1,2,3]. Previous studies have shown that, as an immunosuppressive disease, further infection and organ failure are triggered by a significant decrease in lymphocytes in patients with sepsis [4,5]. Therefore, early diagnosis and treatment of sepsis are essential. With the continuous development of therapeutic tools for sepsis, several biomarkers relevant to the diagnosis, monitoring, and treatment of sepsis have been reported. For example, *TNF*, *IL-1β*, and *IL-6* are three pro-inflammatory factors that play an essential role in sepsis and may serve as potential biomarkers [6].

Regulatory T cells (Tregs) are important as immune cells with strong immunosuppressive effects in tumor-like diseases, infectious diseases, and autoimmune diseases [7,8]. Fabienne Venet et al. found an increased percentage of circulating Tregs in sepsis patients correlated with a decreased proliferative response of lymphoid tissue [9]. Wisnoski et al. used a mouse model of sepsis to find that Tregs directed by *IL-4* contributes to increased susceptibility to sepsis death [10].

Despite these findings, the exact role of Tregs in sepsis and their potential as diagnostic targets remain unclear. The complex interplay between Tregs and other immune cells, as well as the underlying molecular mechanisms, are still not fully understood. This gap in knowledge highlights the need for further research to elucidate the mechanisms by which Tregs contribute to sepsis progression and to identify reliable Treg-related biomarkers for early and accurate diagnosis. With the development of machine learning and deep learning algorithms, including large language models, various models have shown significant advantages in fields such as medical imaging [11,12], omics data mining [13], and biomarker identification [14]. This study aims to fill the gap in understanding the role of Tregs in sepsis and provide potential diagnostic targets for early and accurate diagnosis utilizing multiple models to identify Treg-related biomarkers.

## 2. Materials and Methods

To address the unresolved issues regarding the role of Tregs in sepsis and their potential as diagnostic targets, we conducted a comprehensive analysis of transcriptional data from sepsis patients and controls. We obtained differentially expressed genes (DEGs) through differential expression analysis of transcriptomic data from the GEO database (https://www.ncbi.nlm.nih.gov/geo/) (accessed on 15 August 2023). The calculation of the immune cell infiltration abundance was based on the CIBERSORT algorithm, revealing significant differences in Tregs between sepsis and control groups. We then explored the diagnostic significance of TRGs using various machine learning algorithms to screen DRGs and construct a diagnostic model. Finally, we analyzed the DRG-related drug network using the DGIdb database (https://www.dgidb.org/) (accessed on 15 August 2023) to identify potential therapeutic targets.

### 2.1. Data Acquisition

The internal training and test data used in this study were obtained from the GSE26440 dataset of the GEO database. This dataset includes transcriptomic data from 98 sepsis samples and 32 control samples. We divided the dataset into a training set (80 sepsis samples and 24 control samples) and a test set (18 sepsis samples and 8 control samples) based on a ratio of 8:2. As an external test set, the GSE95233 dataset included transcriptome data from 51 sepsis samples and 22 control samples.

### 2.2. Differential Expression Analysis

In this study, we implemented the limma algorithm based on the R package (4.2.3) “limma”. We input the original gene expression matrix and sample labels into the algorithm, set the threshold to adj. *p* < 0.05 and the absolute value of logFC greater than 0.5, and finally screened out 536 DEGs. The Benjamini–Hochberg test was used to correct *p*-values.

### 2.3. Immune Cell Abundance Calculation

The expression of DEGs was extracted from the original gene expression matrix and then entered into the CIBERSORT algorithm. Then, the final infiltration abundance of 22 immune cells corresponding to all samples was obtained.

### 2.4. Enrichment Analysis

In this study, the KEGG (https://www.genome.jp/kegg/) (accessed on 15 August 2023) and GO (https://www.geneontology.org/) (accessed on 15 August 2023) enrichment analyses of TRGs were performed with the R package (4.2.3) “clusterProfiler”, and the bubble and histogram of the enrichment analyses were plotted based on the R package (4.2.3) “ggplot2”.

### 2.5. Protein Interaction Analysis

To identify the interactions between the intersecting genes, we input the intersecting genes into the String database (https://cn.string-db.org/) (accessed on 15 August 2023) and visualized the PPI network using Cytoscape software (3.10.2). In addition, we applied three algorithms (Degree, EPC, and Radiality) in this software to determine the core genes in the network.

### 2.6. Machine Learning Algorithms Used to Screen Diagnosis-Related Genes and Diagnostic Model Construction

#### 2.6.1. Random Forest

RF is an integrated learning method based on a tree classifier. In this study, we applied a random forest algorithm for gene screening and diagnostic model construction. Specifically, we used the scikit-learn package of python to implement RF to build a diagnostic model. At first, this study applied ten-fold cross-validation to the training set samples for the selection of key parameters (the same strategy was used for other classifiers). The parameter “n_estimators” was set between 100 and 1000, and the parameter “criterion” was set between “gini” and “entropy”. After selecting the best parameters, the final accuracy was obtained in the test set.

#### 2.6.2. Support Vector Machine

Support vector machine is a generalized linear classifier for binary classification that solves the maximum margin hyperplane for the learned samples according to the decision boundary. It can find the optimal classification hyperplane for both classes of samples in the original space when linearly divisible. When linearly indistinguishable, it adds relaxation variables and maps the samples from the low-dimensional input space to the high-dimensional space using a nonlinear mapping to make them linearly divisible so that the optimal classification hyperplane can be found in that feature space. In this study, we applied python’s scikit-learn package to implement SVM to build a diagnostic model. The parameter “kernel” was selected from “linear”, “poly”, “rbf”, and “sigmoid”. The “degree” was chosen within the range of 1 to 3.

#### 2.6.3. Logistic Regression

Logistic regression (LR) is a log-linear model capable of creating regression formulas based on data on categorical boundary lines. The results of the linear model are compressed within [0, 1] using a sigmoid function, which in turn outputs the category probabilities. In this study, we used the scikit-learn package of python (2.6.7) to implement LR to construct a diagnostic model. And the parameter “C” was chosen between 0.1 and 3.

#### 2.6.4. Deep Neural Network

A deep neural network (DNN) is a neural network composed of an input layer, multiple hidden layers, and an output layer. The layers are fully connected. This research used python’s scikit-learn package to implement DNN to build a diagnostic model. The parameter “solver” was selected from “lbfgs”, “Adam”, and “SGD”.

#### 2.6.5. Self-Encoder

An automatic encoder (AE) consists of an encoder and a decoder, both of which are realized by a neural network. In this paper, an AE was implemented based on the keras framework. The specific parameters were set as follows: both the encoder and the decoder were composed of two hidden layers with dimensions of [10, 5] and [5, 10], respectively. The output dimension of the bottleneck layer was 2. The optimizer was set to ‘adam’, the loss function was set to ‘mse’, and the batchsize and epoch were set to 4 and 100, respectively. Finally, the corresponding category with the larger output value of the bottleneck layer was taken as the category judged by the final classifier.

#### 2.6.6. Noise Reduction Self-Encoder

DAE damages some data by adding noise to the input data based on AE and then restores them to the original input data through encoding and decoding. DAE and AE have the same implementation, network structure, and parameter settings. We set its noise coefficient as 0.1, and the noise obeyed a normal distribution with a mean of 0 and a variance of 1.

### 2.7. Drug Network Analysis

In this study, the DGIdb database (https://www.dgidb.org) (accessed on 15 August 2023) was used to predict the potential drugs or molecular compounds interacting with DRGs (Figure S7).

### 2.8. Methods for scRNA-Seq Data Analysis

In this study, we employed the Seurat package (4.2.3) for data preprocessing and normalization. The dataset was loaded using the ‘readRDS’ function, and quality control metrics were visualized with violin plots. Data normalization was performed using the ‘LogNormalize’ method with a scale factor of 10,000. High-variability genes were identified using the ‘vst’ method, and the top 2000 high-variability genes were selected for downstream analysis. Expression data were scaled to ensure that each gene had a mean of 0 and a variance of 1 across all cells. To reduce the dimensionality of the dataset, principal component analysis (PCA) was conducted on the high-variability genes. The first 1500 high-variability genes were used for PCA. The original distribution of cells was visualized using t-distributed stochastic neighbor embedding (t-SNE). To correct for batch effects, the Harmony algorithm was applied, and the corrected data were visualized using PCA and t-SNE plots. Cells were clustered using the Louvain algorithm with a resolution of 1.0, and the optimal number of clusters was determined by evaluating different resolutions. Cell types were annotated using the SingleR package (4.2.3) by comparing the expression profiles of the cells to a reference dataset from the Human Primary Cell Atlas. Annotated cell types were visualized using UMAP and t-SNE plots. Cell–cell communication was analyzed using the CellChat package (4.2.3), which infers communication networks based on ligand–receptor interactions. Communication probabilities were calculated and visualized using circle plots, heatmaps, and bubble plots. Analysis was performed on different cell types to identify key signaling pathways and interactions.

### 2.9. qRT-PCR

Whole-blood samples of ten patients with sepsis and ten healthy people were collected from Beijing Tsinghua Changgung Hospital (Beijing, China). Demographic information can be found in Table S1 of Supplementary Materials. The screening criteria for sepsis patients were based on sepsis 3.0 [1]. Whole-blood samples were obtained from the patients, and the quantitative real-time polymerase chain reaction (RT-PCR) was performed. None of the patients had a history of autoimmune disorders, neoplastic diseases, or oral immunosuppressants. The studies involving human participants were reviewed and approved by the Beijing Tsinghua Changgung Hospital (NCT05095324).

## 3. Results

### 3.1. Differential Expression Analysis and Immune Landscape of Sepsis Transcriptome Data

**Figure 1:**
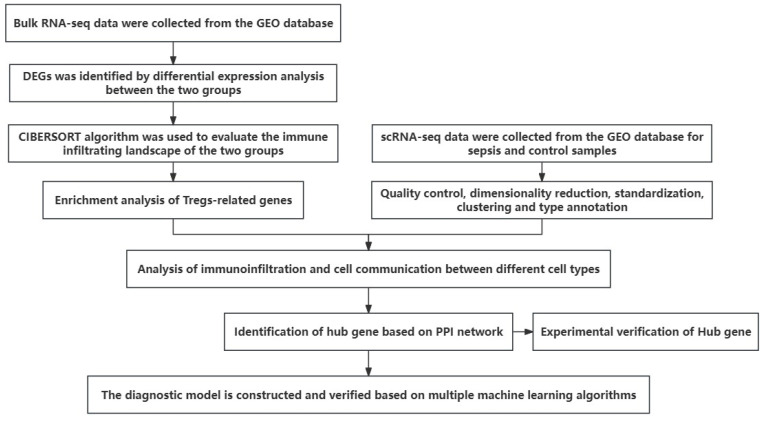
The overall flow chart of the paper.

**Figure 2:**
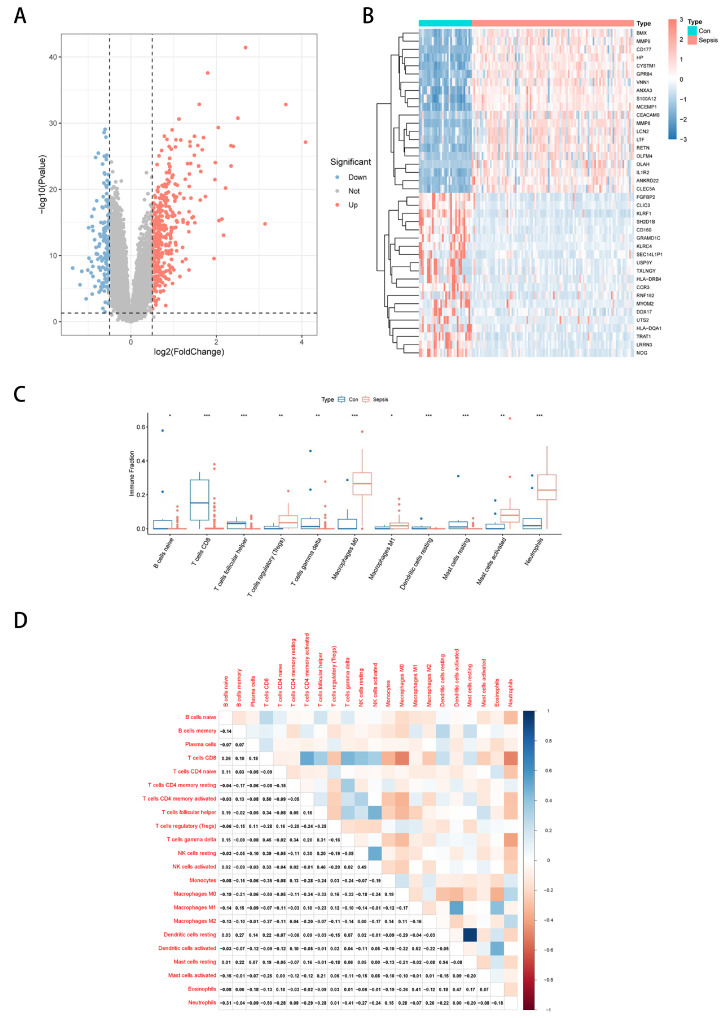
Differential expression analysis and immune infiltration analysis. (**A**,**B**) are heatmaps and volcano maps obtained through differential expression analysis of sepsis and control samples, respectively. (**C**) is the difference box diagram of the infiltration abundance of immune cells in the two groups obtained by the CIBERSORT algorithm. (**D**) is the heatmap obtained through correlation analysis between immune cells. *** represents *p* < 0.001; ** represents *p* < 0.01; * indicates *p* < 0.05.

First, the overall workflow of this study is illustrated in Figure 1. We first performed differential expression analysis on the sepsis transcriptome data from the GSE26440 dataset using the limma algorithm to obtain 536 differentially expressed genes (DEGs). The expression heatmap of the top-ranked DEGs obtained from this analysis in the sepsis and control groups is given in Figure 2A. Figure 2B gives the volcano plot obtained from this analysis, where blue represents significantly downregulated genes, and red represents significantly upregulated genes. We give the correlation of differentially expressed genes in the Supplementary Materials. Further, we employed the CIBERSORT algorithm [15], a computational tool designed to deconvolute the expression profiles of bulk RNA sequencing data into the abundance of various immune cell types. By taking the expression of DEGs as input, CIBERSORT outputs the infiltration abundance of multiple immune cells in both groups. This approach allows us to estimate the relative proportions of immune cell subsets in the tissue samples, providing valuable insights into the immune landscape of the study groups. We give the difference in the infiltration abundance of these immune cells in sepsis and its control group (Figure 2C). The heatmap of the correlation analysis between immune cells is given in Figure 2D. As shown in the figure, Tregs were significantly different in the two groups. We further analyzed the correlation between DEG and Treg abundance. The scatter plot displayed in Figure S1 shows some of the DEGs significantly correlated with Tregs and the rest in the Supplementary Materials. We also give the correlation results for Tregs and DEGs in Supplementary Materials.

### 3.2. Enrichment Analysis and PPI Analysis of Treg-Related Genes

**Figure 3:**
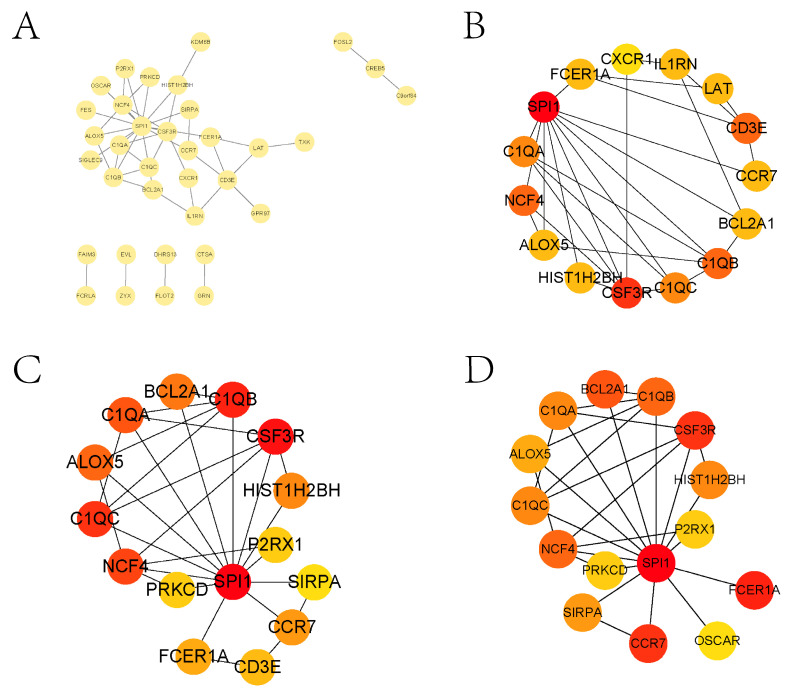
PPI analysis and core gene identification of Treg-related genes. (**A**) is the PPI network of Treg-related genes. (**B**–**D**) are core genes to screen using three algorithms in Cytoscape: Degree, EPC, and Radiality.

The infiltration abundance of Tregs in sepsis and its control group was significantly different, and the abundance of DEGs and Tregs was significantly correlated. To identify potential regulators of Treg function, we collected Treg-related genes based on these correlations and performed Gene Ontology (GO) and Kyoto Encyclopedia of Genes and Genomes (KEGG) enrichment analyses. The significant pathways enriched are displayed in Figure S2A–D (q < 0.05). We found that most of these pathways were closely associated with the development of sepsis and are discussed in detail in Section 4. To further explore the molecular interactions of Treg-related genes, we constructed a Protein–Protein Interaction (PPI) network (Figure 3A). We screened the core genes in the network using three algorithms in Cytoscape software (3.10.2): Degree, EPC, and Radiality (Figure 3B–D). After taking the intersection of the genes selected by the three algorithms, we identified ten core genes: ALOX5, BCL2A1, C1QA, C1QB, C1QC, CCR7, CSF3R, FCER1A, HIST1H2BH, and NCF4. Finally, we validated the expression of these core genes using the external dataset GSE95233 (Figure S3A–J). The expression of all ten genes was significantly different between the sepsis and control groups, supporting their potential roles in the immune response during sepsis.

### 3.3. Construction of Diagnostic Models Based on Multiple Machine Learning Algorithms

**Figure 4:**
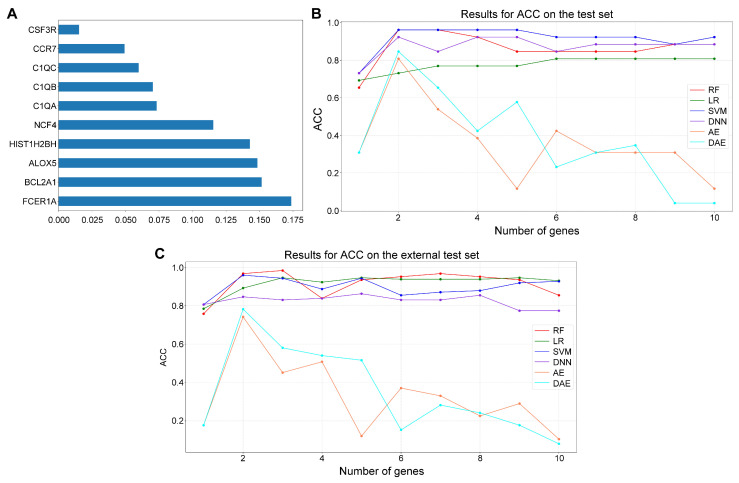
Construction of diagnostic models for core genes using machine learning algorithms. (**A**) is the histogram of the weights of all core genes obtained using the RF algorithm. (**B**,**C**) are the ACC line graphs of the diagnostic models constructed by selecting the top-ranked genes based on the RF, LR, SVM, DNN, AE, and DAE models, respectively.

We first used the RF algorithm to filter the diagnosis-related genes (DRGs) and drew the histogram in Figure 4A. The training set consisted of 104 samples, including 24 control samples and 80 sepsis samples. The testing set included 26 samples, comprising 8 control samples and 18 sepsis samples. We ranked all the features according to their weights, and the higher the weight means, the more influential the feature. Therefore, we applied different classifiers to construct the diagnostic model according to the top one to the top ten features with the highest weight ranking. In the internal test set, the RF classifier achieves 0.9615 for the top two genes, 0.8077 for the top six genes, 0.9615 for the top two genes, and 0.8077 for the top six genes with the SVM classifier. We found that the two encoder-based deep learning methods (AE and DAE) have lower accuracy, which may be due to the fact that a large number of parameters of both encoders are limited by the small sample size and cannot be fully trained (Figure 4B,C). In addition, ten diagnostically relevant genes were validated separately in the internal and external test sets (Figures S4 and S5). All genes had diagnostic significance for sepsis with AUCs greater than 0.7 in both test sets.

### 3.4. Cell Communication Analysis Identifies Key Intercellular Pathways

**Figure 5:**
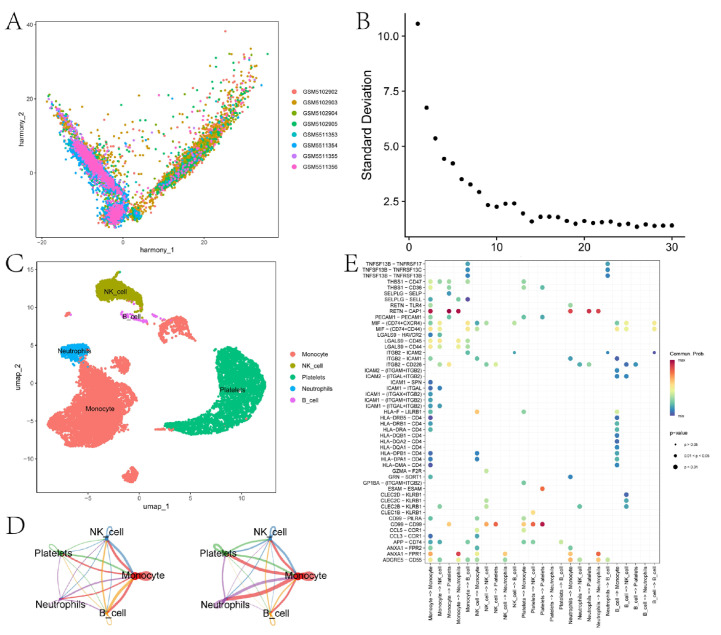
Analysis results of sepsis scRNA-seq data. (**A**) Batch correction of different sepsis samples using the harmony package. The figure shows the distribution of different samples in the dimensions of harmony_1 and harmony_2. (**B**) PCA to identify the standard deviation of different principal components. The x-axis represents the principal components (from 1 to 30), and the y-axis represents the standard deviation. (**C**) Distribution of different cell types in the two-dimensional UMAP space. (**D**) Network diagrams of communication quantity (**left**) and strength (**right**) between different cell types. In the network diagrams, nodes represent cell types, and the thickness of the edges indicates the quantity or strength of communication. (**E**) Bubble plot of significant communication pathways between different cell types. The figure displays the communication pathways between cell types, with the sizes of the bubbles representing the communication probability and the color indicating the significance level.

This section compiles the scRNA-seq data of sepsis and performs fundamental quality control analyses. To correct for batch effects among different sepsis samples, we applied the harmony package (4.2.3) to integrate the data (Figure 5A), thereby minimizing technical variations between samples. Subsequently, we conducted principal component analysis (PCA) to identify the variance explained by different principal components (Figure 5B) and ultimately selected the first eight principal components. We identified five types of cells: platelets, monocytes, NK cells, neutrophils, and B cells (Figure 5C). To visualize the distribution of different cell types in a reduced dimensional space, we utilized Uniform Manifold Approximation and Projection (UMAP) analysis (Figure 5C). We further explored the communication networks among different cell types in sepsis. Figure 5D presents the network diagrams of cell–cell communication quantity (left) and strength (right). To identify significant communication pathways between different cell types, we generated a bubble plot (Figure 5E). Ligand–receptor pairs such as ADGRE5-CD55, ANXA1-FPR1, interactions between HLA-related molecules and CD4, interactions between ICAM1 and ICAM2 with integrins, and interactions between TNFSF13B and the TNFRSF family may play important roles in immune responses and inflammatory processes.

### 3.5. Expression Validation of Diagnosis-Related Genes

**Figure 6:**
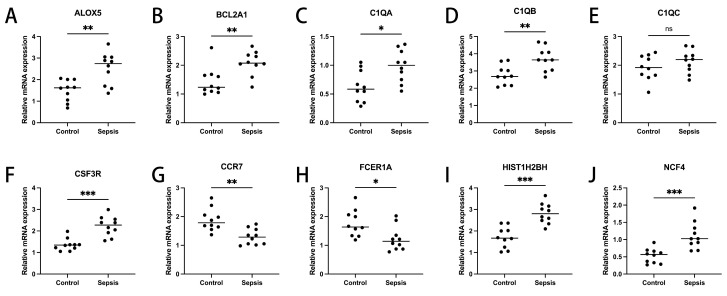
Transcriptional expression in whole blood from sepsis patients and healthy controls of ALOX5 (**A**), BCL2A1 (**B**), C1QA (**C**), C1QB (**D**), C1QC (**E**), CSF3R (**F**), CCR7 (**G**), FCER1 (**H**), HIST1H2BH (**I**), and NCF4 (**J**), relative to GAPDH. Mean ± standard deviation. *** represents *p* < 0.001; ** represents *p* < 0.01; * indicates *p* < 0.05.

To ascertain the expression profiles of ten diagnosis-pertinent genes within the sepsis cohort versus control groups, we utilized quantitative reverse transcription polymerase chain reaction (qRT-PCR). Demographic data pertinent to this study are provided in Supplementary Table S3. Our results demonstrate that, with the exception of C1QC, the expression levels of the remaining genes were markedly distinct between the two cohorts (Figure 6A–J). These findings agree with the outcomes of our bioinformatics analysis. Consequently, further elucidation of the roles these genes play in sepsis holds promise for significant strides in the diagnosis and management of this condition.

## 4. Discussion

Sepsis is a multifaceted syndrome stemming from severe infections. Tregs play a pivotal role in sustaining immune homeostasis and self-tolerance during sepsis, often earning the designation “immune supervisors” [16]. Under normal physiological conditions, Tregs constitute a relatively stable and low-expressing subset, and any disruptions in their homeostasis are typically directly linked to immune dysregulation and immunodeficiencies within the body. To preserve this relative stability, Tregs must orchestrate survival or death signals emanating from cytokines, T cell receptor (TCR)/co-stimulatory signals, and other sources, with signals from dendritic cells (DCs) being a primary contributor [17]. The immune function of Tregs is predominantly upheld by secreting inhibitory cytokines or by repressing the activation and functionality of other immune cells, thereby sustaining immune tolerance and maintaining autoimmune equilibrium within the body. Tregs can directly suppress inflammatory immune responses mediated by activated DCs, B cells, and effector T cells. Our previous studies also revealed that the targeted depletion of Tregs in a sepsis mouse model results in a significant decline in the survival rate of sepsis mice, with mortality predominantly occurring within 48 h post-surgery. This indicates that Tregs play a crucial regulatory role in the early immune homeostasis of sepsis. Additionally, the negative immunosuppressive function of Tregs may be a key factor contributing to immunosuppression in sepsis. In a study on sepsis-induced lung injury, it was observed that following the resolution of pneumonia caused by primary infection, the antigen-presenting ability of DCs significantly diminished. These DCs secreted substantial amounts of TGF-β, leading to massive accumulation of Tregs in the lungs and the formation of an immunosuppressive microenvironment, thereby increasing susceptibility to secondary infections [18]. Patients with sepsis, particularly those with severe sepsis, exhibit heightened vulnerability to secondary infections [16]. Reducing this susceptibility is of paramount importance for the prognosis of sepsis patients. Therefore, we aim to identify sensitive genes of Tregs in sepsis to aid in the diagnosis of sepsis patients and to evaluate the risk of secondary infections in this population.

In this context, we identified several Treg-related genes (TRGs) from the differentially expressed genes between sepsis cases and controls through correlation analysis. Subsequently, we conducted KEGG and GO enrichment analyses on these genes. As illustrated in Figure 4A, biological processes associated with neutrophils and DCs exhibit a strong correlation with sepsis. Neutrophils are the first line of defense of the host against invading pathogens. Peripheral blood neutrophilia during sepsis and complement-mediated neutrophil activation play a paradoxical role in the pathophysiology of sepsis [19,20]. DCs are the most functional specialized antigen-presenting cells of the body, maintaining immune tolerance by migrating to lymph nodes to present their own antigens to lymphocytes in a tolerogenic manner [21]. Astaxanthin can modulate DCs through anti-inflammatory properties to treat sepsis [22]. Upregulation of C-C motif chemokine ligand 2 (*CCL2*) causes renal dysfunction during sepsis [23]. As shown in Figure 4C, Mukhopadhyay S et al. identified functional upregulation of gene expression in human septic shock osteoblast differentiation via transcriptomic meta-analysis [24]. Sepsis is also one of the most common complications and causes of death in patients with alcohol-related liver disease [25].

Moreover, we constructed a PPI network for Tregs using three algorithms to identify the core genes (*ALOX5*, *BCL2A1*, *C1QA*, *C1QB*, *C1QC*, *CCR7*, *CSF3R*, *FCER1A*, *HIST1H2BH*, and *NCF4*) in the network. *ALOX5* is involved in synthesizing leukotrienes, which are potent mediators of inflammation [26]. In addition to inflammatory processes, *ALOX5* is also involved in dendritic cell migration. *ALOX5* can be used as a potential diagnostic biomarker or therapeutic target in sepsis [27]. The *BCL2A1* gene is a direct transcriptional target of NF-κB in response to inflammatory mediators and has good diagnostic and prognostic value in sepsis [28]. *C1qA* deficiency is associated with increased susceptibility to sepsis [29]. A large number of patients surviving sepsis exhibit mental and cognitive impairment, and C1q pathways (*C1qb*, *C1qc*, and *Tyrobp*) are associated with the mental and cognitive impairment observed in the post-sepsis syndrome [30]. Astaxanthin can downregulate *CCR7* expression impeding lipopolysaccharide-induced DC migration, which is beneficial in the treatment of sepsis [31]. *CSF3R* plays a vital role in the proliferation, differentiation, and survival of neutrophil lineage. Fan Y et al. identified diagnostic gene biomarkers such as *FCER1A* using machine learning, which may help in the diagnosis and treatment of patients with septic shock [32], such as *NCF4* in pediatric sepsis patients with abnormal expression [33].

We ranked the importance of ten genes with the RF algorithm and then constructed a diagnostic model for sepsis using multiple machine learning algorithms. Among them, an accuracy of 0.9615 was achieved in selecting the top two genes using the RF classifier. In addition, the AUC of the ten genes was more significant than 0.7 in both the internal and external test sets, which was diagnostic for sepsis. This study constructed a diagnostic model for sepsis based on diagnostic genes. The AUC of this model reached 0.987, which is superior to several existing models (Table S1; Figure S6).

In this study, we employed single-cell RNA sequencing (scRNA-seq) to analyze the cellular landscape of sepsis, with a particular focus on identifying key intercellular pathways involved in the immune response and inflammation. To ensure the robustness of our analysis, we used the harmony package to correct for batch effects among different sepsis samples and selected the first eight principal components based on the results of principal component analysis (PCA). We identified five major cell types—platelets, monocytes, NK cells, neutrophils, and B cells—and visualized their distribution using Uniform Manifold Approximation and Projection (UMAP). Our analysis of cell–cell communication networks revealed significant interactions, including ligand–receptor pairs such as *RETN*-*CAP1* and *ANXA1-FPR1*. The *RETN-CAP1* interaction specifically enhances the Resistin signaling pathway in monocytes via its ligand–receptor pair in sepsis, potentially serving as a diagnostic biomarker for sepsis [34]. *ANXA1* and *FPR1* play a crucial role in the occurrence of adrenal insufficiency by regulating cholesterol ester storage and may represent a novel therapeutic target for maintaining adrenal cortex hormone synthesis in sepsis patients [35].

We validated these genes experimentally; except for *C1QC*, the expression levels of the remaining genes were markedly distinct between the two cohorts. Finally, we also analyzed the drug network of the ten genes (Figure S1 in Supplementary Materials). It is clear from the figure that *ALOX5* and *CSF3R* have interactions with a variety of drugs. For example, Acacetin is a natural flavonoid with a potential protective effect against sepsis [28]. Neulasta (Pegfilgrastim) is indicated to reduce the incidence of infections associated with chemotherapy-induced neutropenia, such as the tendency to reduce the increase in sepsis [36].

In conclusion, our study demonstrates high diagnostic accuracy using machine learning algorithms with a limited set of 10 pre-selected genes. This approach has clear objectives and significant advantages. First, machine learning algorithms, such as random forest (RF) and support vector machine (SVM), can construct diagnostic models with high accuracy and robustness by ranking feature weights and optimizing the model (Supplementary Materials Tables S1 and S2). This provides strong support for the early diagnosis of sepsis. Second, the combination of machine learning and bioinformatics analysis not only validated the differential expression and diagnostic value of these genes in sepsis but also offered new insights into the immune mechanisms underlying sepsis. Additionally, machine learning models demonstrated good generalizability in small sample datasets, effectively handling limited gene sets and maintaining high diagnostic accuracy across different datasets. However, several limitations should be acknowledged. The limited sample size may lead to model overfitting, and the generalizability of our findings across different sepsis subtypes and diverse patient populations remains to be confirmed. Additionally, the interpretability of machine learning models is relatively poor, making it difficult to directly elucidate the biological mechanisms between genes. Future studies should validate the accuracy and robustness of these models in larger and more diverse sample sizes, including different sepsis subtypes and patient cohorts. Integrating causal inference and network analysis could further elucidate the roles of these genes in sepsis and enhance the interpretability of the models. Moreover, prospective clinical trials are needed to translate this model into clinical practice, ensuring its applicability and effectiveness in real-world settings [37,38,39,40].

## 5. Conclusions

In conclusion, this paper analyzed and experimentally validated the role of Treg-related genes in sepsis through PPI networks and various machine learning algorithms. The core genes obtained in this paper can provide a reference for diagnosing sepsis and developing related drug targets in clinical settings.

## Data Availability

The original contributions presented in the study are included in the article/Supplementary Materials. Further inquiries can be directed to the corresponding author.

## Supplementary Materials

The following supporting information can be downloaded at https://www.mdpi.com/article/10.3390/biomedicines13051060/s1: Figure S1: Correlation analysis scatter plots between DEG and Treg abundance are presented. Panels (**A**–**L**) depict the scatter plots showing the significant correlation between the abundance of Tregs and the expression levels of specific DEGs; Figure S2: Enrichment analysis of Treg-related genes. (**A**,**B**) are their GO enrichment analysis’s bar and bubble plots, respectively. (**C**,**D**) are the bar and bubble plots of their KEGG enrichment analysis, respectively; Figure S3: The expression of core genes in the external datasets verifies the box diagram. (**A**–**J**) are the box charts of the expression verification of *ALOX5*, *BCL2A1*, *C1QA*, *C1QB*, *C1QC*, *CCR7*, *CSF3R*, *FCER1A*, *HIST1H2BH*, and *NCF4* in the external datasets, respectively; Figure S4: ROC validation of ten diagnostically relevant genes in the internal test set; Figure S5: ROC validation of ten diagnostically relevant genes in the external test set; Figure S6: The ROC results compared with those of other studies to construct diagnostic models. (**A**–**D**) ROC curves constructed based on diagnostic genes in this paper and references [41,42,43]; Figure S7: DRG drug network analysis; Table S1: ROC results compared with those of other studies to construct diagnostic models; Table S2: ACC changes in algorithms under different noise conditions; Table S3: Demographic information for the sepsis group and the control group.
