# Supplementary material for: Construction of Diagnostic Model for Regulatory T Cell-Related Genes in Sepsis Based on Machine Learning
Source: Biomedicines. 2025 Apr 27;13(5):1060. doi: 10.3390/biomedicines13051060 (PMC12109015; doi:10.3390/biomedicines13051060)
Supplement: Supplementary file 1 [file biomedicines-13-01060-s001.zip › Supplementary Material S1.pdf]

Supplementary Material

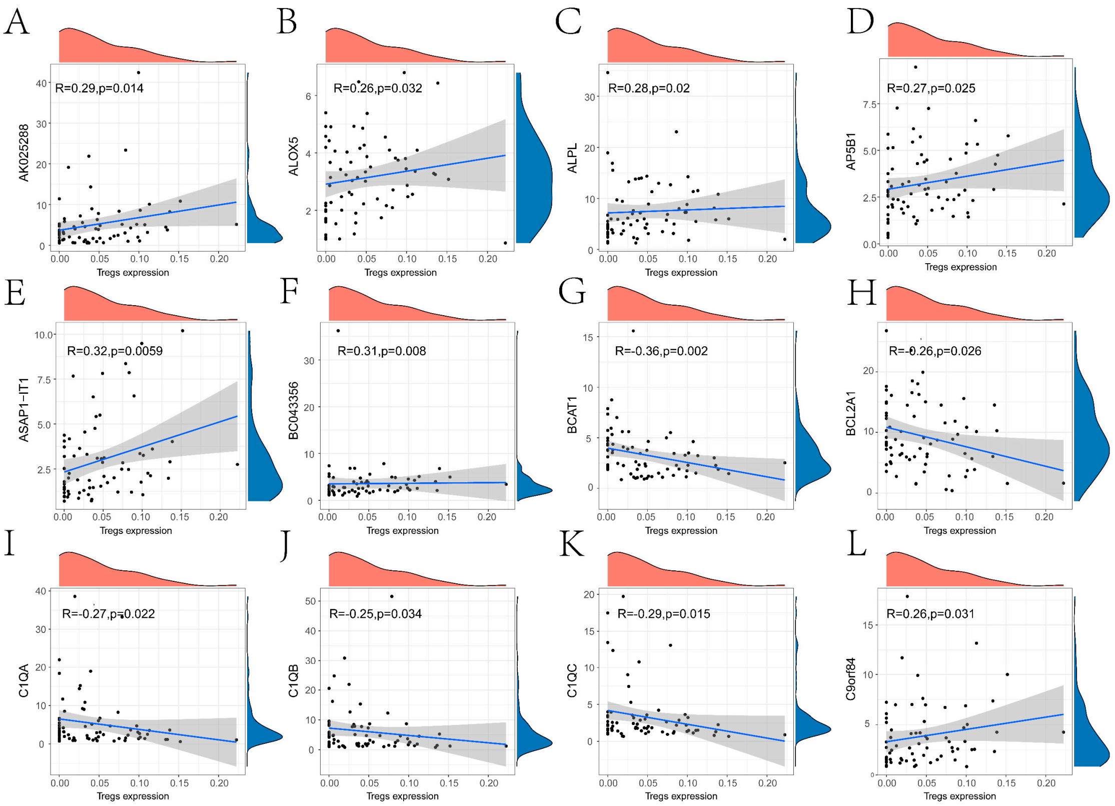

Figure S1. The correlation analysis scatter plots between DEGs and Tregs abundance are presented. Panels A-L depict the scatter plots showing the significant correlation between the abundance of Tregs and the expression levels of specific DEGs.

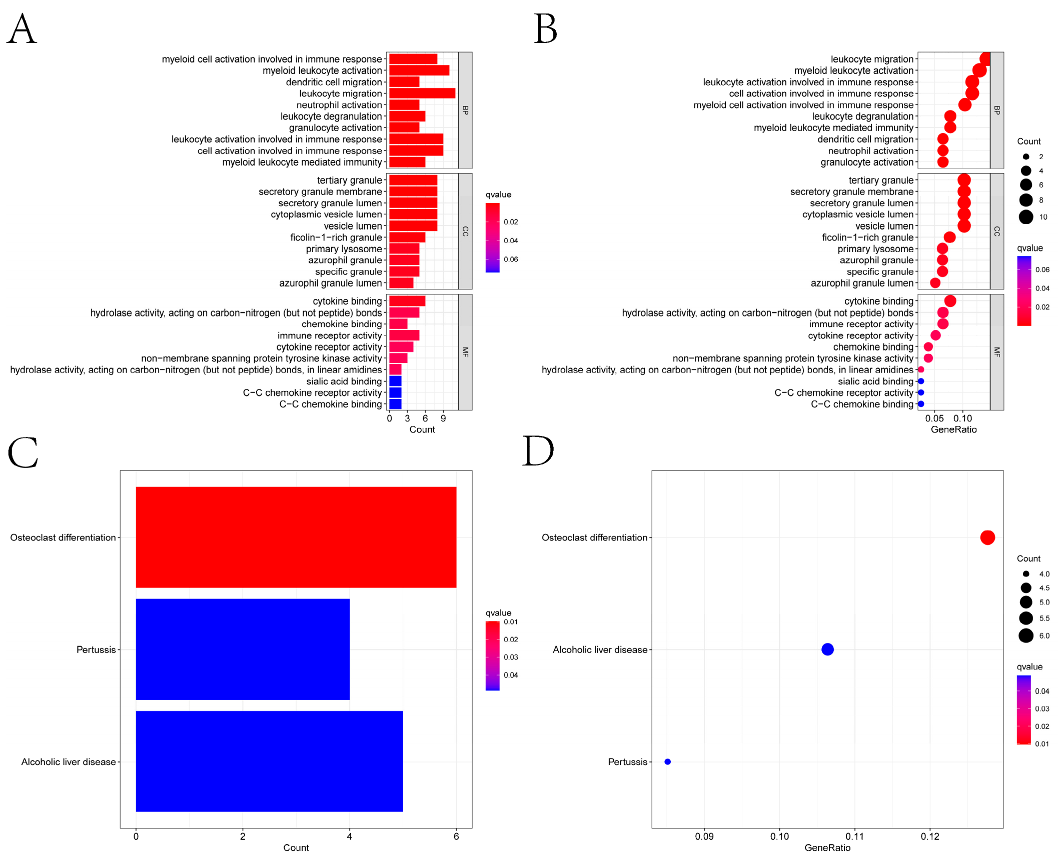

Figure S2. Enrichment analysis of Tregs-related genes. A and B are their GO enrichment analysis's bar and bubble plots, respectively. C and D are the bar and bubble plots of their KEGG enrichment analysis, respectively.

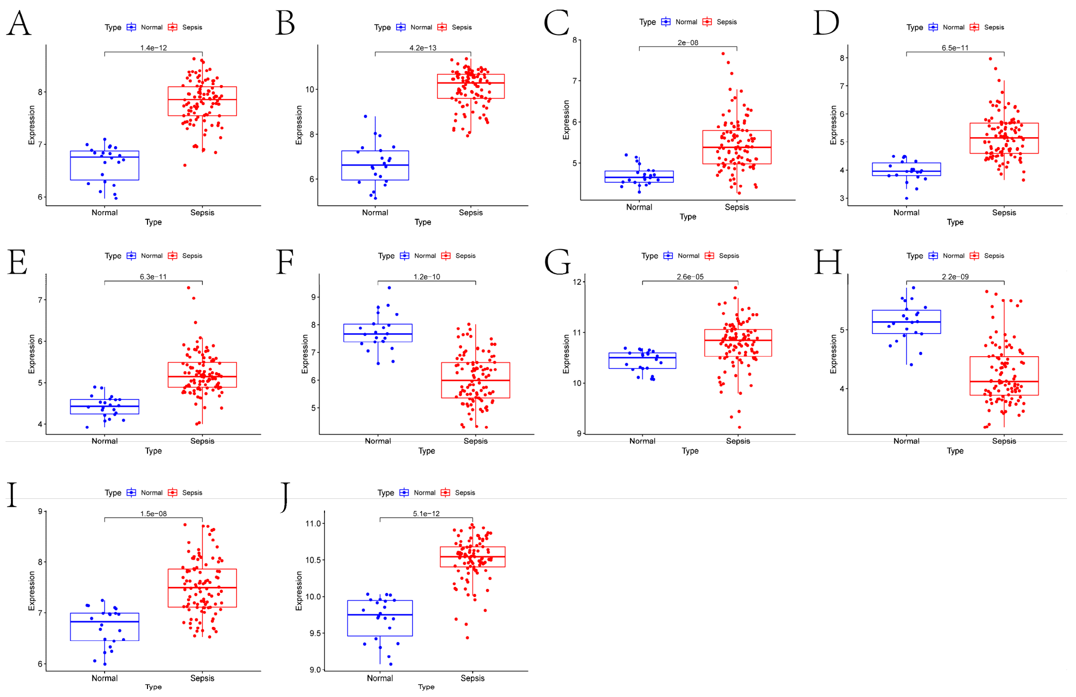

Figure S3. The expression of core genes in external data sets verifies the box diagram. Among them, A-J is the box charts of the expression verification of *ALOX5, BCL2A1, C1QA, C1QB, C1QC, CCR7, CSF3R, FCER1A, HIST1H2BH*, and *NCF4* in external data sets, respectively.

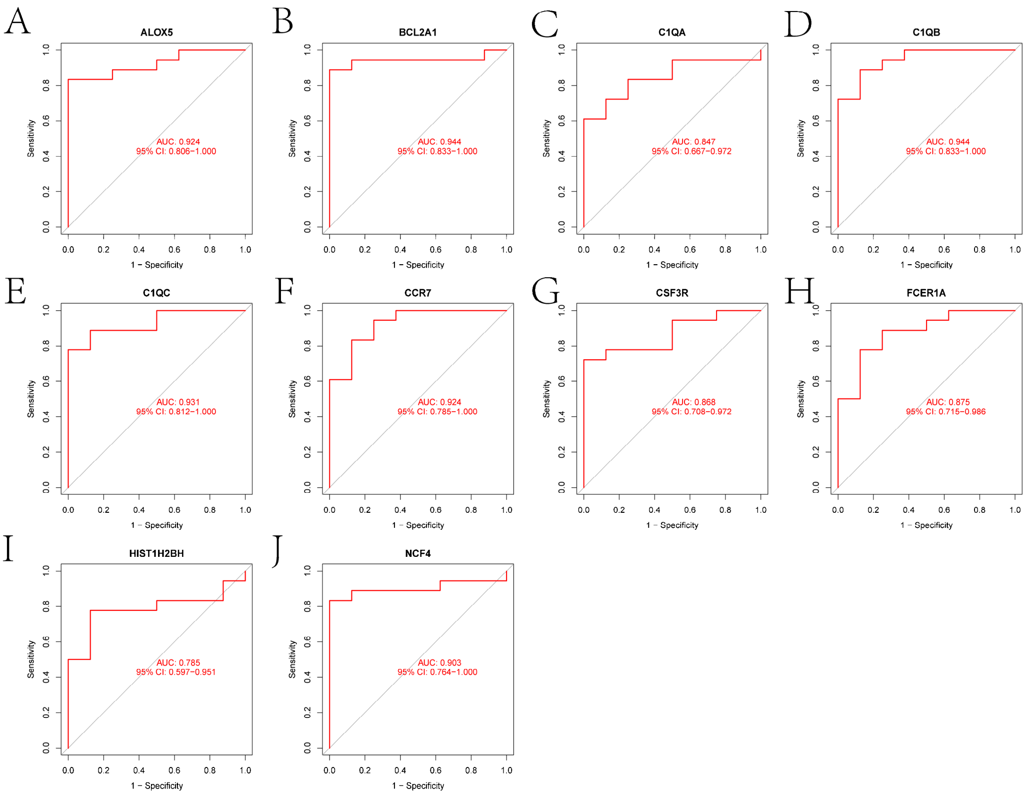

Figure S4. ROC validation of ten diagnostically relevant genes in the internal test set.

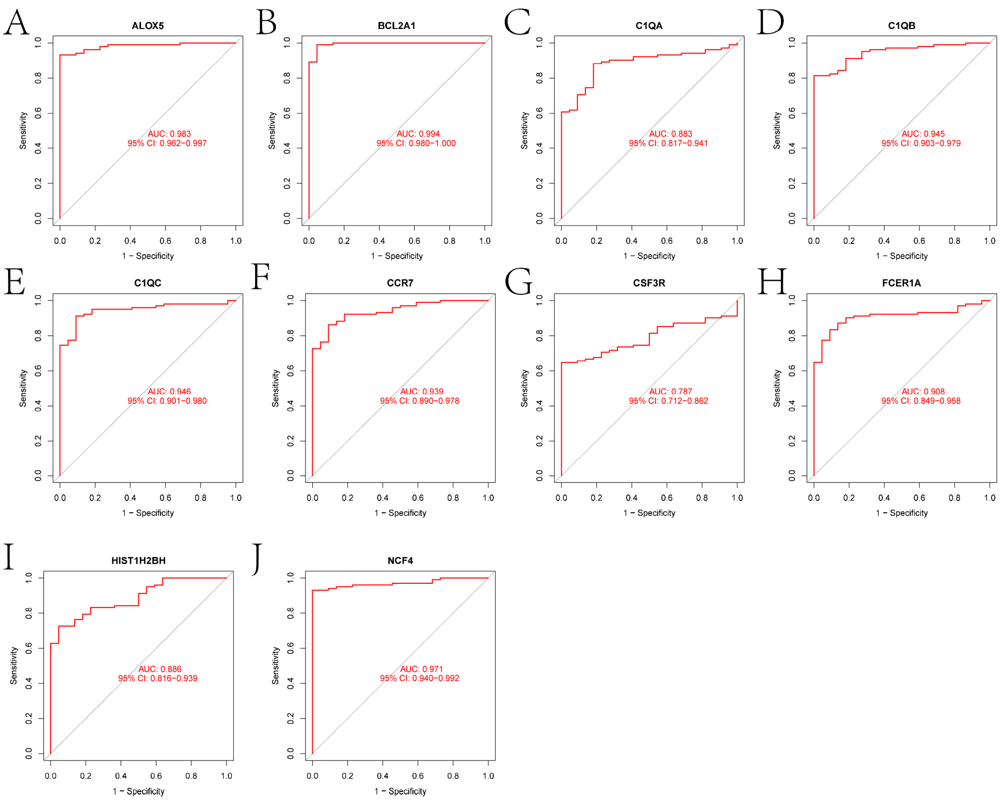

Figure S5. ROC validation of ten diagnostically relevant genes in the external test set.

Tabel S1 The ROC results were compared with other studies to construct diagnostic models

Model	AUC	Sensitivity	Specificity	CI
The model proposed	0.987	0.969	0.939	0.973-1
Literature [1]	0.768	0.813	0.653	0.670-0.865
Literature [2]	0.776	0.75	0.724	0.683-0.870
Literature [3]	0.854	0.781	0.847	0.788-0.919

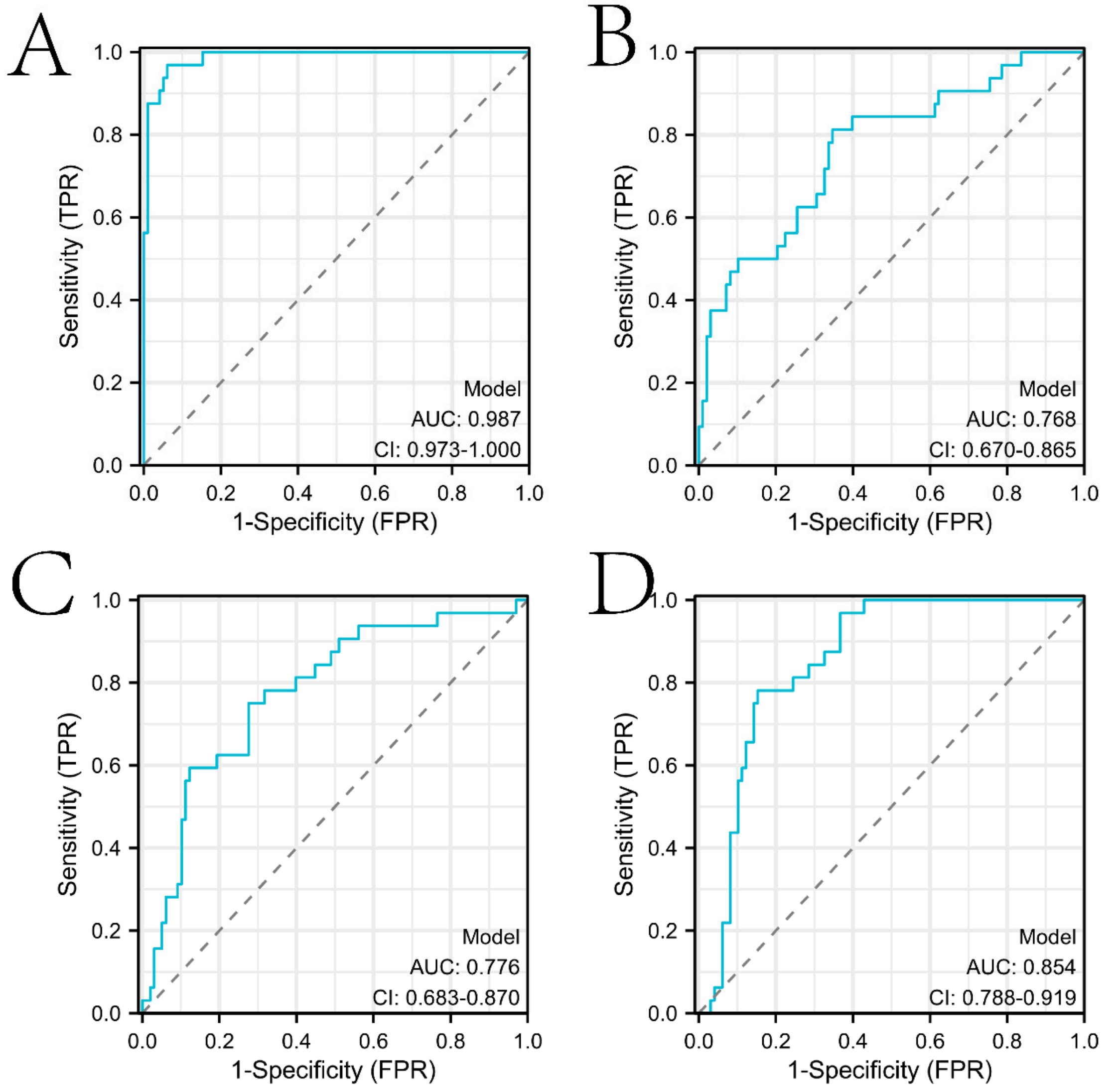

Figure S6. The ROC results were compared with other studies to construct diagnostic models. (A)-(D) ROC curves constructed based on diagnostic genes in this paper, references [1], [2] and [3].

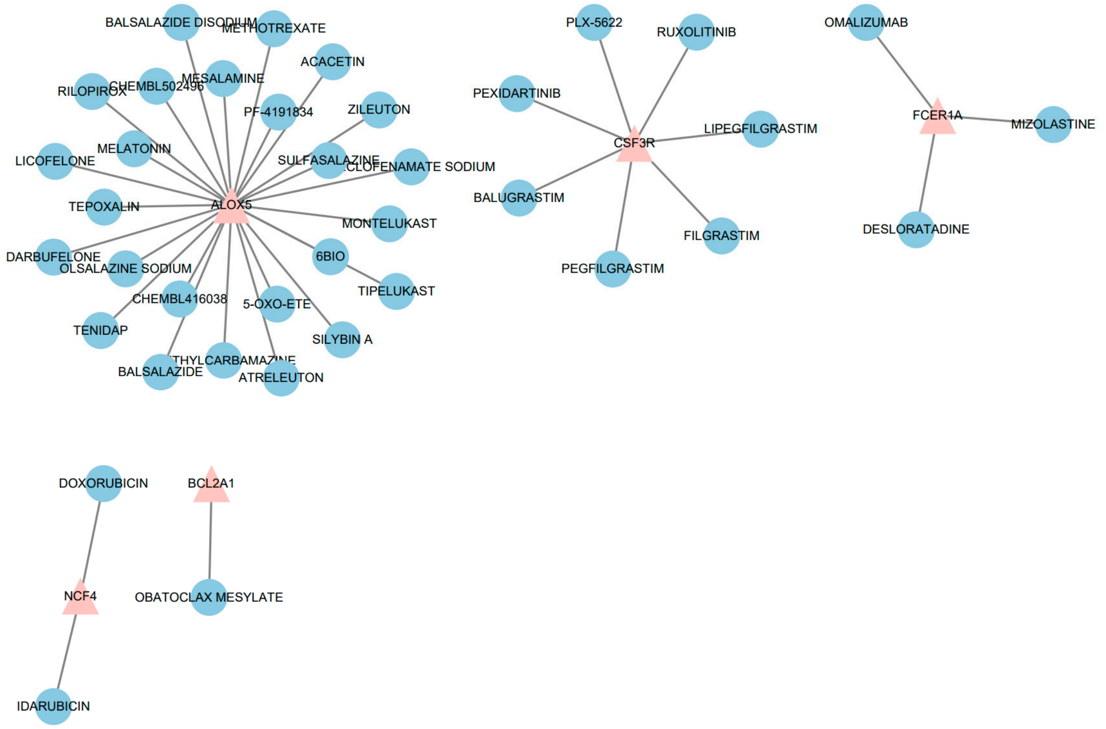

Figure S7. DRGs drug network analysis

1.1 Analysis of Algorithm Noise Resistance

In this study, to evaluate the robustness of the algorithm in the presence of data fluctuations, we introduced Gaussian noise of different levels into the gene expression data. Specifically, we used the Python programming language and its NumPy library to generate Gaussian noise with a mean of 0 and standard deviations of 0.1 and 0.5, respectively, to simulate varying degrees of data fluctuations. The shape of the noise was consistent with that of the original gene expression data, meaning that each gene and sample expression value was added with an independent noise value. Subsequently, the generated noise was added element-wise to the original gene expression data to obtain the noisy data. After adding the noise, no additional normalization or standardization was performed on the data to preserve the impact of the noise on the original data. By this method, we generated independent noisy datasets for each noise level. Table S2 presents the ACC of the algorithm on the test set under different noise levels.

Table S2 ACC changes of algorithms under different noise conditions

Model	Noise level = 0.1	Noise level = 0.5
AE	0.808	0.769
DAE	0.769	0.692
DNN	0.885	0.884
LR	0.808	0.769
RF	0.962	0.923
SVM	0.962	0.923

Table S3. Demographic information for the Sepsis group and the Control group

Parameters	Control	Sepsis	*P*
**Gender**			0.653
Male	6	5	
Female	4	5	
**Ages**			0.001
≥60	1	7	
18-60	9	3	
≤18	0	0	
**Infection Source**			N
Lung	0	6	
Urinary tract	0	1	
Skin or Soft tissues	0	0	
Other	0	3	
**Comorbidities**			0.001
Yes	0	9	
No	10	1	
**ΔSOFA Scores**			0.001
0-2	10	0	
≥2	0	10	
**Lactate levels[a] (mmol/L)**	0.61±0.23	4.09±1.89	0.001

[a] Lactate levels: Mean ± standard deviation, measurement time point: Sepsis group: within 24h after diagnosis of sepsis; Control group: the day of physical examination.

References

[1] Xu, J., Zhu, M., Luo, P., & Gong, Y. (2024). Machine Learning Screening and Validation of PANoptosis-Related Gene Signatures in Sepsis. *Journal of inflammation research*, *17*, 4765–4780. https://doi.org/10.2147/JIR.S461809

[2] Chen, Z., Wei, S., Yuan, Z., Chang, R., Chen, X., Fu, Y., & Wu, W. (2024). Machine learning reveals ferroptosis features and a novel ferroptosis classifier in patients with sepsis. *Immunity, inflammation and disease*, *12*(5), e1279. https://doi.org/10.1002/iid3.1279

[3] Zhao Q, Xu N, Guo H, Li J. Identification of the Diagnostic Signature of Sepsis Based on Bioinformatic Analysis of Gene Expression and Machine Learning. *Comb Chem High Throughput Screen*. 2022;25(1):21-28. doi:10.2174/1386207323666201204130031
